# Functional random forests for curve response

**DOI:** 10.1038/s41598-021-02265-4

**Published:** 2021-12-17

**Authors:** Guifang Fu, Xiaotian Dai, Yeheng Liang

**Affiliations:** Department of Mathematical Sciences, SUNY Binghamton University, Vestal, NY 13850 USA

**Keywords:** Genetics, Genetic association study

## Abstract

The rapid advancement of functional data in various application fields has increased the demand for advanced statistical approaches that can incorporate complex structures and nonlinear associations. In this article, we propose a novel functional random forests (FunFor) approach to model the functional data response that is densely and regularly measured, as an extension of the landmark work of Breiman, who introduced traditional random forests for a univariate response. The FunFor approach is able to predict curve responses for new observations and selects important variables from a large set of scalar predictors. The FunFor approach inherits the efficiency of the traditional random forest approach in detecting complex relationships, including nonlinear and high-order interactions. Additionally, it is a non-parametric approach without the imposition of parametric and distributional assumptions. Eight simulation settings and one real-data analysis consistently demonstrate the excellent performance of the FunFor approach in various scenarios. In particular, FunFor successfully ranks the true predictors as the most important variables, while achieving the most robust variable sections and the smallest prediction errors when comparing it with three other relevant approaches. Although motivated by a biological leaf shape data analysis, the proposed FunFor approach has great potential to be widely applied in various fields due to its minimal requirement on tuning parameters and its distribution-free and model-free nature. An R package named ’FunFor’, implementing the FunFor approach, is available at GitHub.

## Introduction

Functional data analysis has become an active research topic as technological measurement devices become more sophisticated (e.g., new sensors and electrodes). Its far-reaching applications include biological shape and contour studies, global climate change, drug sensitivity curve for cancerous cell lines, functional near infrared spectroscopy (fNIRS), functional magnetic resonance imaging (fMRI), electroencephalography (EEG), population kinetics of plasma folate, and more^1–3^. Our work is driven by a leaf morphology (shape) trait that possesses tremendous variability in nature^4,5^, reflecting ecological and evolutionary drivers^6,7^. Each leaf shape is described as a 910-dimensional functional data object with regular and dense grids. Scientists are particularly interested in exploring which genes truly regulate the variation of the dynamic trajectory of biological shape phenotypes and in predicting shape for future observations. Shape has been found to be affected by gene–gene interactions^8,9^; however, interactive variables are hard to detect because they may have very small marginal/main effects. Manolio et al. summarized a few difficulties in modeling complex traits, including the low power to detect gene-gene interactions^10^.

A typical definition of functional data is to collect data by machines over a dense and regular grid of time/location points, as our motivated leaf shape data does. Despite the fact that the data is measured in the form of multiple discrete points in various aforementioned applications, an underlying trajectory is clearly manifested as a smooth random function. Therefore, modeling the response as a single entry and capturing its dynamic trajectory reflects the true need. Challenges come from the potentially hidden structure and that the data’s distribution is messier than any assumptions. Nonlinear and non-additive relations, which truly reflect the characteristics of the real world’s messy dataset in practice, have been neglected by many existing approaches.

In this article, we propose a new approach named Functional Random Forests (FunFor), which facilitates an extension from the traditional random forests methodology (RF for short; designed for a univariate response) to a functional or curve response setting. The inputs of the FunFor method will be a curve response along with a set of scalar predictors for each observation. FunFor outputs the predicted curve response for each observation along with the importance rank of each predictor according to its association strength with the curve response. The FunFor approach has many beneficial properties: effectively accommodating both the linear and nonlinear associations; jointly modeling multiple predictors instead of isolating them; capturing the intricate higher-order interactions without the need of specifying the order or structure of interactions; being flexible for various predictor types, such as binary, categorical, and/or continuous predictors; demonstrating feasibility for high dimensionality when the number of predictors is larger than the number of observations; embracing a nonparametric approach without assuming any specific model structure or certain distribution; and requiring few tuning parameters. We demonstrate that the FunFor approach performs well through eight simulation settings under various difficulty levels and also one real data analysis. Although the proposed FunFor approach was driven by a genetic leaf shape problem, it has far-reaching potential to be applied widely in various fields that have functional traits^2,3,11^, as the traditional random forest approach does for univariate traits. An R package named ’FunFor’, implementing the FunFor approach, is available at GitHub (https://github.com/xiaotiand/FunFor). It can be installed by running devtools::install_github (“xiaotiand/FunFor”).

While the literature based on the functional linear regression model is vast^12–20^, we want to emphasize that they propose a parametric model structure and assume a linear relationship without focusing on interactions or complex structures. As commented by Chen et al., few methods for simultaneous variable selection and prediction exist in the functional/curve data literature^16^. Additionally, functional analyses exploring higher-order interactions and nonlinear associations without any model structure or distribution assumptions are even rarer, despite the fact that such scenarios widely exist in practice. In general, nonparametric data-driven tree-based approaches have been found to frequently outperform the classic regression approaches when the datasets have more complex structures than general assumptions^21^.

A few attempts have been made to extend the tree-based models from a univariate response to a curve response. Segal assumed three well-known covariance structures^22^: independence, compound symmetry, and a first-order autoregressive model. As pointed out by Zhang and Singer^23^, Segal’s studies were restricted to longitudinal data having a given auto-covariance structure. Abdolell et al. extended the regression tree from a univariate response to a longitudinal response, but they only considered a prognostic predictor^24^. In summary, these early works represented breakthroughs in extending standard regression tree models from one univariate response to a curve response. However, they either mainly focused on the longitudinal data instead of functional data or built only one single tree. The restriction of using one single tree has been already observed^25^. Fu et al. built multiple individual models by applying the traditional random forests approach to fit each individual principal component score of the curve separately without truly incorporating the functional nature of the curve response as a whole unit^8^. Moller et al. used a functional random forests approach to model functional predictors but a scalar response^26^, the opposite of the setting we consider.

In this article, we compare the proposed FunFor approach with three other directly comparable works: the ‘refund’ R package implementing the function-on-scalar regression model with LASSO penalty proposed by Reiss et al.^13,27^, the ‘splinetree’ R package that was built based on the work of Yu and Lambert^11,28^, and the functional random forests (FRF) approach proposed by Rahman et al.^3^. See Scheipl for a comprehensive CRAN task view: functional data analysis^29^. As demonstrated in the “Simulation studies” section, the FunFor approach achieves the most effective and robust performance among the four approaches in the three simulation examples. In addition to the empirical comparisons, we also comment on the common and different technique points of these four approaches. The four approaches can all be utilized for not only detecting important associations between scalar predictors and the curve response but also predicting dynamic trajectory of the curve response.

The differences are as follows: The function-on-scalar regression model shrinks unimportant predictors to zero and is feasible for variable selection in the case of $p>n$ when incorporating the LASSO penalty. However, it relies on a parametric model structure and linear relationship assumptions^13^, without focusing on nonlinear associations and complex structures. Yu and Lambert realized the advantage of functional trees to cope with complex and nonlinear relationships. However, they proposed a multivariate regression tree to fit the coefficients of the spline basis functions or the first few principal component scores of the curve response. Actually, the multivariate modeling does not utilize the time course of the functional data and therefore cannot describe the overall time trajectory of the response in the functional sense. Yu and Lambert gave an example in which applying a multivariate regression tree model to a curve response was not successful^11^. Rahman et al. also proposed a functional random forests approach and obtained good results when applying it to predict the dose-response curve for cancerous lines^3^. There exists multiple detailed technique differences between the two methods: Rahman et al. divided the entire curve into several regions and computed the overall error by summing up several individual errors (i.e. node costs) obtained from each of the regions. The node cost computation at each region is very close to that of the traditional random forest model by using one point (mean or medium of multiple points) per region. However, this region-wise approach may not truly incorporate the functional nature of the response during the tree construction process. Additionally, they did not provide a well-justified rule for how to determine the number of regions. After investigating multiple options, we noticed that the choice of the number of regions greatly affected the results. The characteristics of the function were reflected in their last step by adopting a linear combination of B-spline functions at the leaf nodes to predict the curve response^3^, but they neglected the roles of the covariance structure, which were found to produce incorrect variance estimates^30,31^. Conversely, the FunFor approach integrates the functional nature of the response everywhere into an indispensable system. In addition, the FunFor approach continues to update curve estimates by computing both the mean and the complex covariance structure after the observations are recursively divided into two child nodes. Truly integrating the functional process during both the tree building-up process and leaf node prediction process is not only important but is more accurate.

## Methodology

Let $Y_{ik}, i=1,\ldots ,n;\,k=1,\ldots ,K$ denote the dense but discretely recorded curve response for the $i\text{{th}}$ observation, measured at the $k\text{{th}}$ time (or location) point. Here, *K* is the number of time points, which is set to be the same for each observation; *n* is the number of observations, and *p* is the number of predictors. Let ${\varvec{X}}_i \in {\mathbb {R}} ^ {1 \times p}$ be the $i\text{{th}}$ row of the design matrix, and $X_j, j=1,\ldots ,p$ be the $j\text{{th}}$ predictor.

Technically speaking, the FunFor approach incorporates the following multiple steps: it utilizes the nonparametric functional principal component analysis (FPCA) skills to estimate the underlying random function for each subject; adopts smoothing techniques to approximate the infinite-dimensional mean functions and auto-covariance functions; incorporates the estimated and smoothed function of each observation into a regression tree framework in order to build a functional regression tree; grows an ensemble of functional regression trees; finally, outputs the variable importance measure for each predictor and the predicted curve response for each subject.

### Functional principal component analysis

The observed response data can be modeled as an underlying function plus noise,

1 $$\begin{aligned} Y_{ik}=f_{i}(t_k)+\varepsilon _{ik},\,i=1,\ldots ,n;\,t\in [0,1], \end{aligned}$$

where $f_{1}(t),\ldots ,f_{n}(t)$ are a collection of independent realizations of a random functional process *f*(*t*) and are defined on $L^2([0,1])$. The $\varepsilon _{ik}$ are the independent experimental errors with $E(\varepsilon _{ik})=0$ and $Var(\varepsilon _{ik})=\sigma ^2_{k}$, k=1,...,K. The mean function of *f*(*t*) is $E(f(t))=\mu (t)$, a smooth function of $t\in [0,1]$ and the auto-covariance function of *f*(*t*) is $G(t',t)$, a bivariate positive definite smooth function of $t',t\in [0,1]$. In order to model the auto-covariance function, FPCA interprets $G(t',t)$ as the kernel of a linear integral operator on the space of square-integrable functions on [0, 1], mapping $f\in L^2([0,1])$ to $A_{G} f \in L^2([0,1])$, defined by $(A_{G} f)(t)=\int _0^1 f(s)G(s,t)ds.$ We assume that the operator $A_{G}$ has a sequence of smooth orthonormal eigenfunctions $v_{l}(t)\in L^2([0,1])$ satisfying $\int _0^1 v_{k}(t)v_{l}(t)dt=\delta _{kl}$ (here $\delta _{kl}$ is the Kronecker symbol), with ordered eigenvalues $\lambda _{1}\ge \lambda _{2}\ge \ldots \ge 0$.

The functional principal component expansion decomposes the random functions $\{f_i(t)\}$ as^31,32^

2 $$\begin{aligned} f_{i}(t)=\mu (t)+\sum _{l=1}^{\infty }\zeta _{il}v_{l}(t), \end{aligned}$$

where the sum is defined in the sense of $L^2$ convergence and $\zeta _{il}=\int _0^1 (f_{i}(t)-\mu (t))v_{l}(t)dt$ are uncorrelated random variables with $E(\zeta _{l})=0$, and $var(\zeta _{l})=\lambda _{l}$. Here, $\zeta _{l}$ is frequently referred to as the $l\text{{th}}$ functional principal component score (PC). The curve response can be estimated as^17,33,34^

3 $$\begin{aligned} {\tilde{f}}_i(t)={\hat{\mu }}(t)+\sum _{l=1}^{L}{\hat{\zeta }}_{il}{\hat{v}}_{l}(t), \,i=1,\ldots ,n; \,t \in [0,1]. \end{aligned}$$

Here, ${\hat{\mu }}(t)$ is the estimated overall mean function and ${\hat{v}}_{l}(t)$ and ${\hat{\zeta }}_{il}$ are the estimated eigenfunctions and estimated functional PCs of the estimated auto-covariance function ${\hat{G}}(t',t)$, respectively. *L* is the number of PCs to be retained, which is pre-specified according to the proportion of total variation of the curve response explained by the first few PCs. The ${\tilde{f}}_i(t)$ obtained from Eq. (3) is an estimate of the random function $f_i(t)$.

### Smoothing and estimating

Since the real data are contaminated with measurement errors, the sample mean vector $\bar{{\varvec{Y}}}$ and eigenvectors of the sample covariance matrix $\bar{{\varvec{G}}}$ (a $K\times K$ matrix) of the raw data tend to be noisy, affecting the accuracy of the results. Therefore, we need to perform smoothing processes to obtain the estimates ${\hat{\mu }}(t)$ and ${\hat{G}}(t',t)$, all of which are contained in Eq. (3). Specifically, we applied the univariate P-spline smoother approach to get an estimate of the mean function ${\hat{\mu }}(t)$. To obtain an estimate of the covariance function, ${\hat{G}}(t',t)$, we applied the sandwich smoother and the fast covariance estimation (FACE) algorithm^35,36^. The sandwich smoother is a bivariate smoothing approach that employs a tensor product structure to simplify asymptotic analysis and speed up computation. It applies univariate P-spline smoothers simultaneously to both the row and column coordinates of the sample covariance matrix. Situations where $K>500$ raise challenges for covariance matrix smoothing in terms of vast computational and storage burdens for high-dimensional matrices. Therefore, FACE, a fast implementation of the sandwich smoother^36^, was developed for high-dimensional covariance function smoothing used in functional data analysis.

### Functional regression tree

In this step, we input the estimated smooth function ${\tilde{f}}_i(t)$ obtained from Eq. (3) described in “Functional principal component analysis” and “Smoothing and estimating” sections into the functional regression tree. A functional regression tree is constructed by successively splitting the predictor space into mutually exclusive subregions and then repeatedly partitioning observations into those subregions^37^. Let *s* denote a possible split-point value of a given predictor, $X_j$. Let *R* denote a parent node under consideration. For each *j* and each *s* combination, a pair of left and right descendent child nodes are defined as, $R_L(j,s)=\{({\varvec{X}}_i, {\tilde{f}}_i(t))|X_{ij}<s\} \,\,\text {and} \,\,R_R(j,s)=\{({\varvec{X}}_i, {\tilde{f}}_i(t))|X_{ij}\ge s\}.$

Inspired by the univariate splitting criterion, we designed a new splitting function for a curve response. We first define the average function of all smooth functions that are divided into the node *R* as ${\hat{f}}_R(t) = \sum _{i \in R} {\tilde{f}}_i(t)/n_R$. Here, $n_R$ is the number of observations divided into the node *R*. Accordingly, the new residual sum of squares (RSS), defined for the functional curve at a node *R*, will be computed based on the integrated squared error (ISE)^38^

4 $$\begin{aligned} RSS_f(R) = \sum _{i \in R} \int _{0}^{1} [{\tilde{f}}_i(t) - {\hat{f}}_R(t)]^2 dt. \end{aligned}$$

The new splitting criteria, measuring the between-node separation, can be defined as

5 $$\begin{aligned} \phi (j,s,R) = \int _{0}^{1} [{\hat{f}}_{R_L(j,s)}(t)-{\hat{f}}_{R_R(j,s)}(t)]^2 dt. \end{aligned}$$

A fixed number of split-points are scanned for each predictor under consideration, and the top split-point (the one that maximizes $\phi (j,s, R)$) is ultimately chosen to produce a split. Specifically, we scan all possible combinations of split-points for each categorical predictor and scan ten equally spaced split-points for each continuous predictor.

During the tree-growing process, the estimated curve response ${\tilde{f}}_i(t)$ in Eq. (3) for each observation will keep updating at each candidate split of each node. This is because a split causes a regrouping, so the observation pool (and also the mean and covariance functions) in the parent node differs from those of its descendant nodes. When a tree grows deeper, the homogeneity of the curve response in each leaf node will increase. A fully-grown functional regression tree may be too complex, likely overfitting the data and leading to poor prediction accuracy. We first grow the tree to the maximum depth and record the sequence of splits. We then prune the tree back in reverse order^37^. When a tree is pruned, we calculate the average integrated squared error of the corresponding subtree through fivefold cross-validation. Finally, we determine the optimal functional regression tree by using the subtree with the lowest prediction ISE.

### Randomness

Similar to the traditional RF approach, the FunFor approach introduces two instances of randomness and also grows an ensemble of functional regression trees to yield a consensus vote. First, instead of using all original observations, the data used to grow each tree is from one bootstrap sample of the original observations. Aggregating predictions over hundreds of trees can significantly reduce variance and increase both prediction accuracy and stability, when compared to a single tree. Secondly, the method gains additional prediction accuracy by randomly drawing only a subset of predictors to determine the best split for each parent node of each tree; hence the predictors which have strong main effects or strong correlations do not always dominate. In addition, when the number of predictors is very large, the consideration of only a randomly-selected subset of variables for each split greatly improves computational efficiency. In the end, we predict the curve response of the $i\text{{{th}}}$ observation as ${\hat{f}}_i(t)=\bar{{\hat{f}}}_R(t)$, where $\bar{{\hat{f}}}_R(t)$ is the average of ${\hat{f}}_R(t)$ predicted at the leaf nodes across multiple trees that contain the $i\text{{{th}}}$ observation through bootstrap sampling.

### Permutation variable importance measure

The output of the FunFor is the predicted curve response for each observation, ${\hat{f}}_i(t)$, along with the permutation variable importance measure (PVIM) for each predictor based on its association strength with the curve response. The PVIM of each predictor $X_j$ is obtained via the difference in average prediction error before and after randomly permuting $X_j$ while keeping other predictors untouched^39^. Let $B_q$ denote the set of out of bag (OOB) samples of the $q\text{{{th}}}$ tree and ‘ntree’ denote the total number of trees, then the PVIM of predictor $X_j$ is defined as

6 $$\begin{aligned} PVIM(X_j)= \frac{1}{ntree}\Bigg (\sum _{q=1}^{ntree} \frac{1}{|B_q|} \sum _{i\in B_q} \left[\int _{0}^{1} [{\tilde{f}}_i(t) - {\hat{f}}_i^p(t)]^2 dt - \int _{0}^{1} [{\tilde{f}}_i(t) - {\hat{f}}_i(t)]^2 dt \right]\Bigg ), \end{aligned}$$

where ${\hat{f}}_i(t)$ is still the predicted curve response obtained from the FunFor approach without permuting $X_j$, ${\hat{f}}_i^p(t)$ is the predicted curve response after permuting $X_j$ and $|B_q|$ denotes the size of the set. The larger a PVIM is, the more important the corresponding predictor is in predicting the curve response. A predictor with a negative or close-to-zero PVIM value is interpreted as unimportant^39^. The PVIM assesses each predictor’s impact without isolating all other predictors nor requiring interactive terms to be explicitly added into the model.

### Tuning parameters

There are two tuning parameters involved in the FunFor approach. The first tuning parameter is the size of the subset of the predictors that are considered at each split, designated ‘mtry’. For a univariate response, it has been suggested that ‘mtry’ should be approximately one third of the total number of predictors^40^. We use a ‘mtry’ value of 40% (close to *p*/3) and it works well for all of the eight simulation settings and the real data analysis. Another tuning parameter is the value of *L* described in Eq. (3). We adopt two options to determine the value of *L*. Option 1: We apply the most standard rule in the functional data analysis literature and use all of the observations at the root node. Then we fix this value of *L* at all splits during the tree growth process. As claimed by Di et al.^41^, this rule simultaneously satisfies two criteria: the proportion of total variation explained by the first *L* principal components is larger than a predetermined threshold (e.g., 90%) and the proportion of variation explained by any additional principal component is less than another predetermined threshold (e.g., 1%). This rule works well in their simulation studies and practical analyses as demonstrated by Di et al.^41^. Option 2: Specify the minimum percentage of total variation explained by the data (e.g., 90%). We then adaptively and flexibly determine and update the value of *L* at each split according to different observations obtained at each child node.

## Simulation studies

We experiment with eight simulation settings to explore the performance of the FunFor approach in detecting the true predictors under various levels of difficulty. Each simulation design is performed with 100 replications. The details of Simulation Examples 1–5 are elaborated in the following subsections, and Simulation Examples 6–8 can be found in the Supplementary Appendix. The tuning parameters are set as $L=3, mtry = 0.4 p$, and $ntree = 100$ when applying the FunFor approach to fit each of the eight simulation examples. The FunFor approach is compared with three other relevant approaches. We choose their default settings for the tuning parameters when applying the ‘refund’ and ‘splinetree’ packages and divide the *K* time points into 10 regions when implementing the FRF Matlab code provided in the github. Three criteria are used to assess not only the variable selection but also the prediction accuracy of the four approaches under comparison.

1. *M* is defined as the minimum selection size that is required to detect all of the true predictors in each replication. We calculate multiple quantiles and also the average of *M* across the 100 replications. The closer the different quantiles are to the total number of true predictors, the robuster the approach is in variable selection.
2. *Rk* is defined as the average rank of each true predictor across 100 replications. The smaller the *Rk* and the average *M* values are, the more effective the approach is in variable selection.
3. MAE is defined as the mean absolute error^42^, which is the same rule used to assess the prediction performance as Rahman et al. did^3^. The smaller the MAE is, the more accurately the approach predicts.

### Simulation Example 1: a single-true-predictor design

In this design, we consider a scenario in which only one true predictor is associated with the response and mimic the motivated real dataset, i.e., the gene-shape association. Please see the Supplementary Appendix for three extra simulation examples that also mimic the real dataset but with increasing difficulty levels. The genetic marker data is coded as 0 for aa, 1 for Aa, and 2 for AA. As a categorical variable, each genetic marker is generated using a binomial distribution with a random minor allele frequency ($\text {MAF} \sim \text {Uniform}(0.1, 0.5)$). Among all generated genetic markers, we randomly choose one marker to truly connect with the response (call it $X_1^*$) and set all the rest to be noise. We set three 360-dimensional curves as the mean responses, $\varvec{\mu _{1}}$, $\varvec{\mu _{2}}$, and $\varvec{\mu _{3}}$, under each of the three genotype categories of the true genetic marker. These three mean curves are used to describe three true poplar leaves with varying shapes. See Fu et al. for the shape analysis details used to transform an image to a curve^43^.

Then the shape curve samples for each replication are generated based on the genotype of this true genetic marker and the three true leaf shape curves as

7 $$\begin{aligned} {\varvec{Y}}^T_i= [\varvec{\mu _{1}}^T, \varvec{\mu _{2}}^T, \varvec{\mu _{3}}^T]\cdot I_{X_1^*} + \varepsilon _{360 \times 1}, \,\,i=1,\ldots ,100, \end{aligned}$$

where $\varepsilon _{360 \times 1} \sim {\mathcal {N}}({\varvec{0}}_{360 \times 1}, \Sigma _{360\times 360})$, and $\Sigma _{360\times 360}$ is the empirical sample covariance matrix of the three true leaf shapes. Here, $I_{X_1^*}$ is an indicator function that is defined as

$$I_{X_1^*} = {\left\{ \begin{array}{ll} (1,0,0)^T, &{} \text{ if } \text{ the } \text{ genotype } \text{ of } X_1^* \text{ is } \text{ AA, } \\ (0,1,0)^T, &{} \text{ if } \text{ the } \text{ genotype } \text{ of } X_1^* \text{ is } \text{ Aa, } \\ (0,0,1)^T, &{} \text{ if } \text{ the } \text{ genotype } \text{ of } X_1^* \text{ is } \text{ aa. } \end{array}\right. }$$

In this simulation, we set $n = 100, p = 100,$ and $K= 360$. As shown in Table 1, the results of Simulation Example 1 achieve a level of perfection because it ranks the true predictor as 1.01 on average; additionally, there is no diversity or mistakes among the 100 replications judging from its various quantiles of *M*.

**Table 1:** Results of the Simulation 1.

Simulation 1	$Rk ^\text{{{a}}}$	$M_{5\%} ^\text{{{b}}}$	$M_{25\%}$	$M_{50\%}$	$M_{75\%}$	$M_{95\%}$
$X^*_{1}$	1.01	1	1	1	1	1

$^\text{{{a}}}$ *Rk* stands for the average rank of the single true predictor across 100 replications. The smaller the *Rk* value is, the more effective the approach performs in variable selection.

$^\text{{{b}}}$ *M* stands for the minimum selection size that is required to obtain the single true predictor in each replication. The closer the different quantiles is to the total number of the true predictors, the robuster the approach is in variable selection.

**Figure 1:**
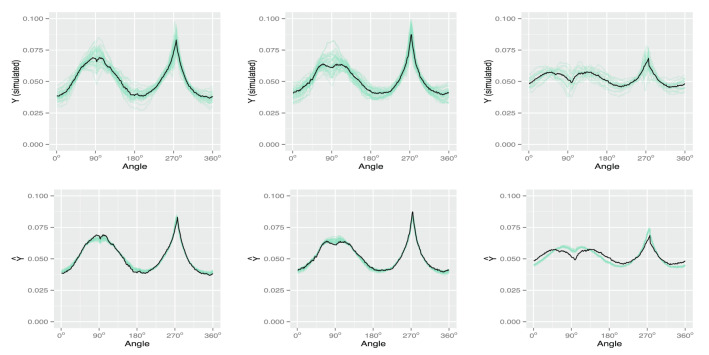
Visualization of the prediction performance of the FunFor approach for one replication in the Simulation 1: The top panel illustrates the simulated response samples (transparent green lines on the first row) and the bottom panel demonstrates the predicted curve response (transparent green lines on the the second row) output from the FunFor approach. Each column corresponds to a different genotype category. The black lines demonstrate the three true shapes used for generating data.

**Figure 2:**
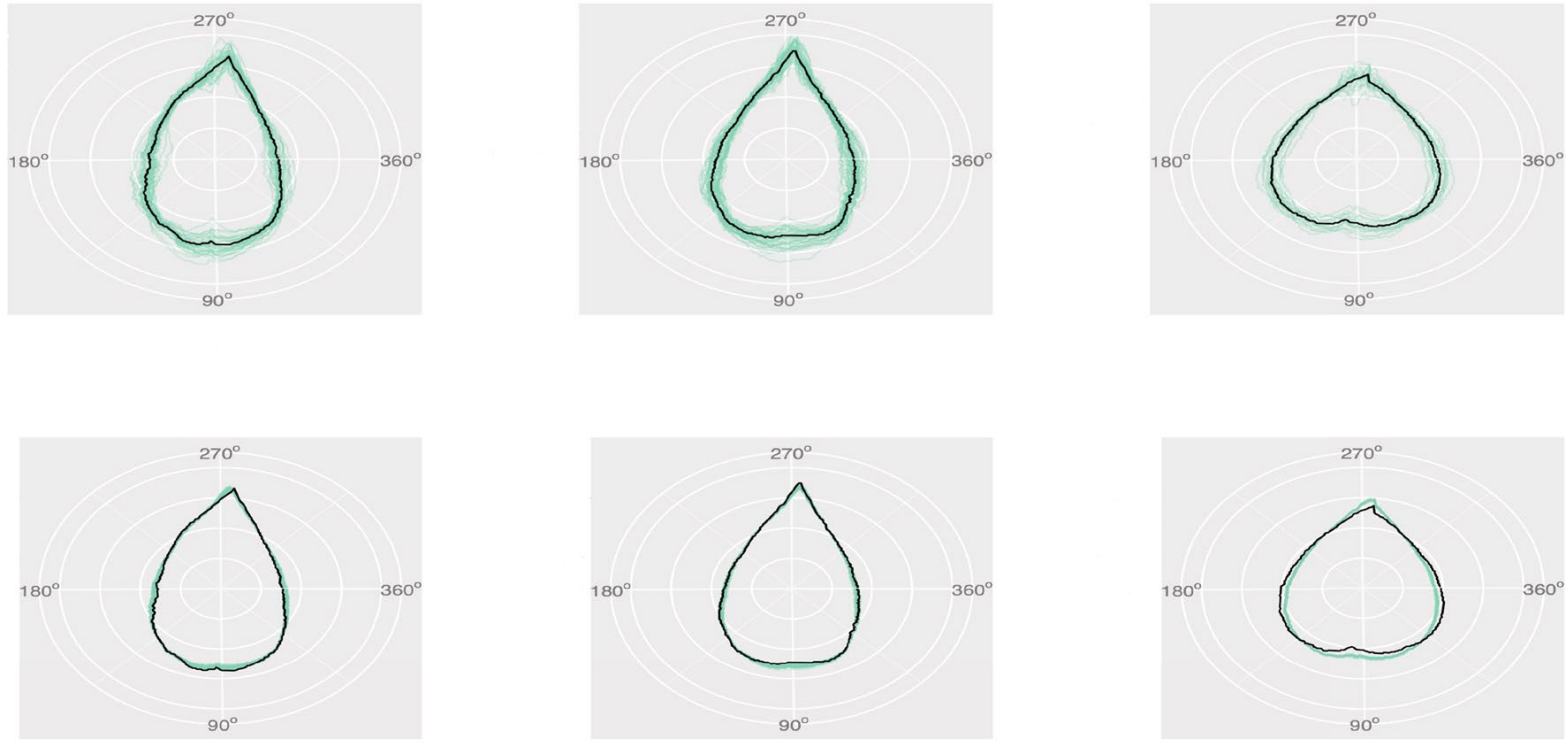
Same information as the Fig. 1, but converting curves into shapes.

In addition to the variable selection results, we also demonstrate the prediction performance of the FunFor approach by visualizing one replication that is randomly chosen from Simulation 1 (see Figs.  1, 2). Figure 2 displays information that is similar to that in Fig. 1, but converts the curves back to shapes. The 100 simulated samples are divided into three categories based on the genotype information of the single true genetic marker $X_1^*$. Each separate column of Figs.  1 and 2 represents a different genotype. The top panel of Figs. 1 and 2 stands for the original simulated response samples $Y_{ik}$ and the second row is the predicted response curve output from the FunFor model, i.e., ${\hat{f}}_i(t)$. The true leaf used to generate data is visualized by a solid black line and the samples are demonstrated by transparent green lines. As shown in Figs. 1 and 2, the predicted curve responses are very close to the truth, with predictions capturing not only global trajectories but also subtle local details of the shape. Despite the fact that the simulated response samples are noisy (see the first rows of Figs.  1, 2) and the 99/100 predictors are generated to be confounding noise, the prediction results visually look great. In the following simulations, we will formally investigate the prediction accuracy using the MAE value.

### Simulation Examples 2–4: a statistical model design

In this design, we simulate data through statistical models so that we can easily control the parameter values, difficulty levels, association structures, and also compare with other relevant approaches. The time points $t_{k}$ for each observation are scheduled to be $\{1,\ldots , K\}$, which is consistent with the focused regular and dense setting. The predictor variables, *X*s, are generated from $N({\varvec{0}}, \Sigma _X)$, where $\Sigma _X$ follows an *AR*(1) design ($\sigma =1, \rho = 0.6)$ so that the joint effects and correlations between predictors (as those exist in the practices) can be incorporated. The coefficient functions are set to be

$$\begin{aligned} \beta _1 (t) = \text {sin}(20 \pi t), \,\, \beta _2 (t) = \text {cos}(20 \pi t),\,\, \beta _3 (t) = \text {cos}(20 \pi t) + \text {sin}(20 \pi t). \end{aligned}$$

The random error $\varvec{\varepsilon }_i=\{\varepsilon _{i1},\ldots ,\varepsilon _{ik},\ldots ,\varepsilon _{iK}\}$ is generated from $N({\varvec{0}}, \Sigma _\varepsilon )$, where $\Sigma _\varepsilon$ follows *AR*(1) design with $(\sigma _k=(5 \cos (k) + rnorm(K, 0, 1)) / 10, \rho = 0.01)$ to take their auto-correlation into account.

#### Simulation Example 2

The response curve and the two true predictors ($X_2, X_3$) and their interactive terms ($X_2X_3$) are connected as

$$\begin{aligned} Y_{ik} = X_{i2} \beta _1(t_{k})+ X_{i3}\beta _2(t_{k}) + X_{i2}X_{i3}\beta _3(t_{k}) + \varepsilon _{ik}. \end{aligned}$$

In this simulation, we set $n = 100, p = 100,$ and $K= 500$.

#### Simulation Example 3

This Simulation Example 3 has the same design and model as the Simulation Example 2, except we change the parameters to $n = 200, p = 500,$ and $K= 100$ to explore the robustness of the approach under a different parameter setting.

#### Simulation Example 4

The response curve and the true predictors are connected as

$$\begin{aligned} Y_{ik} = X_{i2} X_{i3} \beta _1 (t_{k})/3 + \varepsilon _{ik}. \end{aligned}$$

In this simulation, we remove the main/individual effects and keep only one interactive term, thus increasing the difficulty level. In this simulation, we set $n = 100, p = 100,$ and $K= 100$.

The results of Simulation Examples 2–4 for the four approaches under comparison are demonstrated in Tables 2 and 3. The FunFor approach performs the best in most of the cases among the four approaches in terms of both variable selection and prediction accuracy. As one can see from Table 2, the FunFor approach consistently ranks the two true predictors among the top three ones on average no matter if main effects exist (Simulation 2) or not (Simulation 4). When the *p*/*n* ratio increases to be 2.5 times higher, it still effectively ranks the two true predictors among the top five ones on average (Simulation 3). For Simulations 3, the ranks of the FunFor approach are slightly worse than those of the refund and FRF. However, these two approaches perform much worse in Simulation 4 when there are no main/individual terms included in the model. Additionally, their ranks in Simulation 4 are more than five times higher than those in Simulations 2–3. We are not surprised at this gap because lack of strong main/individual effects or lack of ability in detecting complex structures has been a common challenge for many approaches. In addition, the prediction accuracies (i.e., MAEs) of the FunFor approach are consistently the smallest among the four approaches in all of the three simulation settings. Table 3 demonstrates the quantiles of the minimum selection size for the four approaches in order to simultaneously detect all of the true predictors. We can see that the FunFor approach is the most robust approach no matter if the main/individual effects exist or not. The *M* values of the FunFor approach do not show much variation from 5 to 95%, which further indicates that the majority of replications effectively achieve the best results under various settings. The FRF and refund also perform great under Simulation 3 without being affected by the *p*/*n* ratio, but their performances greatly decrease under the more complex scenario in Simulation 4.

**Table 2:** The variable selection and prediction performances of the four approaches for the Simulation Examples 2–4.

Simulation	Method	Average of $M^\text{{{a}}}$	${Rk_{X_2^*}}^\text{{{b}}}$	$Rk_{X_3^*}$	$\hbox {MAE}^\text{{{c}}}$
Simulation 2	FunFor	2.58	2.01	2.12	1.48
	FRF	6.70	4.37	4.08	2.29
	Splinetree	21.91	20.88	21.91	2.30
	Refund	2.00	1.18	1.82	2.23
Simulation 3	FunFor	5.17	4.66	4.68	1.50
	FRF	2.83	2.40	1.51	2.28
	Splinetree	38.58	37.40	38.42	2.28
	Refund	2.00	1.58	1.43	2.31
Simulation 4	FunFor	3.74	2.25	3.06	0.58
	FRF	25.35	15.02	17.65	0.62
	Splinetree	56.99	43.49	40.35	0.62
	Refund	28.40	16.26	18.10	0.62

^a^The average of *M* is the average of the minimum selection size that requires to obtain all of the true predictors across 100 replications. The smaller, the better.

$^\text{{{b}}}$*Rk* stands for the average rank of each true predictor across 100 replications. The smaller, the better.

$^\text{{{c}}}$MAE is the prediction error. The smaller, the better.

**Table 3:** The quantiles of the minimum selection sizes of the four approaches for the Simulation Examples 2–4.

Quantiles of $M^\text{{{a}}}$	Method	$5\%$	$25\%$	$50\%$	$75\%$	$95\%$
Simulation 2	FunFor	2.00	2.00	2.00	3.00	4.00
	FRF	2.00	2.00	4.00	9.00	18.00
	Splinetree	16.00	19.00	22.00	24.00	28.00
	Refund	2.00	2.00	2.00	2.00	2.00
Simulation 3	FunFor	3.00	4.00	5.00	6.00	7.05
	FRF	2.0	2.0	2.0	3.0	5.1
	Splinetree	32.00	35.00	38.00	41.00	46.05
	Refund	2.00	2.00	2.00	2.00	2.00
Simulation 4	FunFor	2.00	2.00	3.00	3.00	5.05
	FRF	3.00	12.00	22.50	36.25	62.05
	Splinetree	16.00	38.00	58.00	81.00	94.00
	Refund	2.00	2.00	2.00	98.00	100.00

$^\text{{{a}}}$*M* stands for the minimum selection size that is required to include all of the true predictors in each replication. The closer of different quantiles, the robuster.

### Simulation Example 5: a null model

To fully verify that the good performances of the FunFor approach are not caused by chance, we generate data from a null model, under which there is no association existing between the curve response and any of the predictors. That is, the $X\hbox {s}$, random errors, and $\beta _1(t)$ are all set as above, but we do not connect *Y* with any of the predictors. Specifically, the response is generated as

$$\begin{aligned} Y_{ik} = \beta _1 (t_k) + \varepsilon _{ik}. \end{aligned}$$

**Figure 3:**
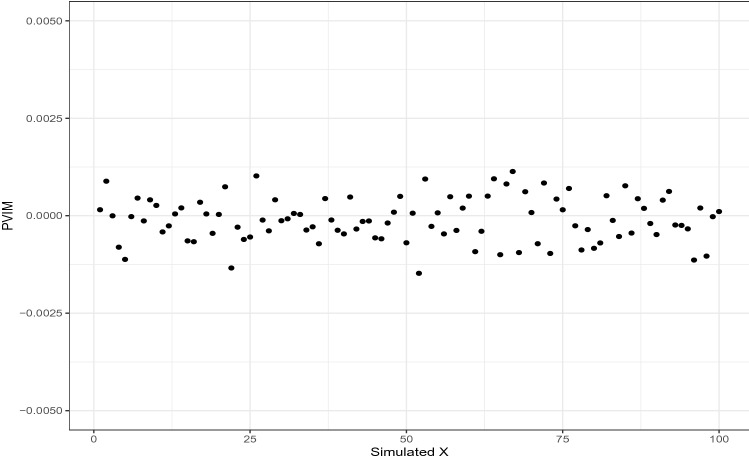
Average prediction variable importance scores across 100 replications for all of the predictors output from the FunFor approach in Simulation 5.

In this simulation, we set $n = 100, p = 100$, and $K = 100$. As demonstrated in Fig. 3, the average PVIM scores across 100 replications for all of the predictors output from the FunFor approach are close to zero, locating within a very small range of [− 0.0025, 0.0025]. It’s good to see that no predictor stands out, which is well aligned with the true situation.

## Real data analysis

**Figure 4:**
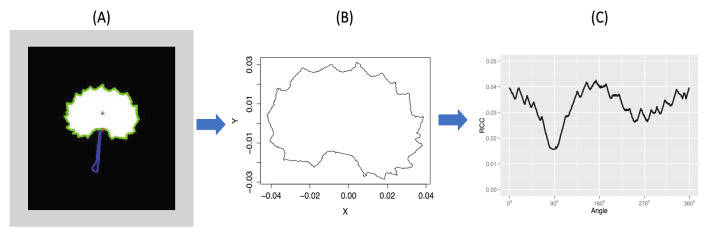
The shape curve generation process: recognize a shape outline demonstrated from (**A,B**); and transform a shape to a curve demonstrated from (**B,C**).

In this section, we analyze the leaf shapes of a natural population of 421 *Populus euphratica* (also named *Euphrates Poplar* or *Desert Poplar*) trees, which naturally grow along river valleys in arid regions of the Xin Jiang province of China. Twenty-five leaves were randomly collected from each tree. The leaf shapes of *Populus euphratica* are polymorphic with complex subtle details on their boundaries, so a small set of loose landmark points will be incapable of accurately describing them. Therefore, we use a $910 \times 1$ dimensional curve to describe each shape based on the directional radii method^44^. As observed in Fig. 4, the one-to-one mapping between the shape and the 910-dimensional curve is accurate, with sharp and complex teeth on the boundary also being well-maintained. Then we perform the alignment to filter out the variations caused by pose (translation, size, and rotation) before performing the analysis^43,44^. The directional radii curve then becomes standard functional data measured on dense and regular grids. The scientific interest is to detect which genes are truly associated with the variation of the trajectories of biological leaf shape traits, and to predict morphology for future observations. Since the twenty-five leaves collected from the same tree are correlated and share the same genetic information, we use the average of the twenty-five shape curves as the curve response of each observation. For each of the 421 trees, 104 markers were also genotyped.

We empirically explore whether the FunFor approach is robust over a few different choices of tuning parameters. Since ‘mtry’ is suggested to be $p/3\approx 35$, we employ three *mtry* values: 10, 20, and 35. In addition, we also try the two options, the fixed version and the adaptive version, for choosing *L*. For the fixed setting, we choose $L=4$ because the first four PCs can explain about 90% of the total variation in the curve response using all of the observations at the root node. The performance of FunFor approach is assessed by evaluating the average prediction integrated squared error between the observed and the predicted curve response using five-fold cross-validation. Judging from the results demonstrated in Table 4, the prediction ISE does not differ much among the six different combinations, which empirically verifies the robustness of the FunFor method over reasonable choices of the two tuning parameters. In the following, we interpret the results obtained from the adaptive *L* setting and $mtry = 20$ for the real data analysis because it achieves a relatively optimal prediction ISE.

**Table 4:** The prediction error obtained from the fivefold cross-validation under six combinations of the two tuning parameters.

$L^\text{{{a}}}$	$mtry^\text{{{b}}}$		
	10	20	35
Adaptive *L*	119.5	118.9	119.4
Fixed $L = 4$	119.7	119.3	118.9

$^\text{{{a}}}$*L* is the number of functional PCs to be retained in Eq. (3).

$^\text{{{b}}}$*mtry* is the size of the subset of the predictors that are considered at each split.

**Figure 5:**
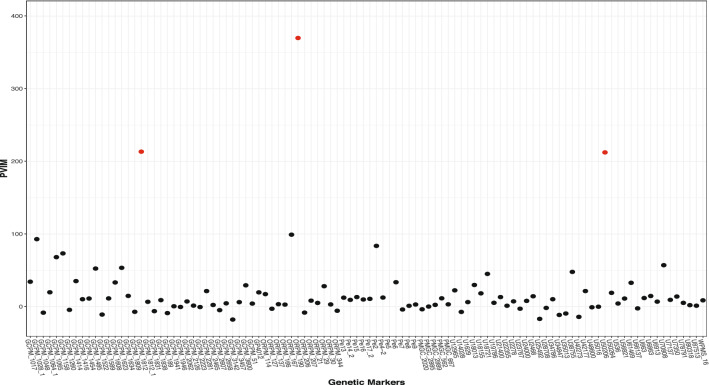
Prediction variable importance measures of 104 genetic markers. The highest PVIMs are highlighted with red dots.

**Figure 6:**
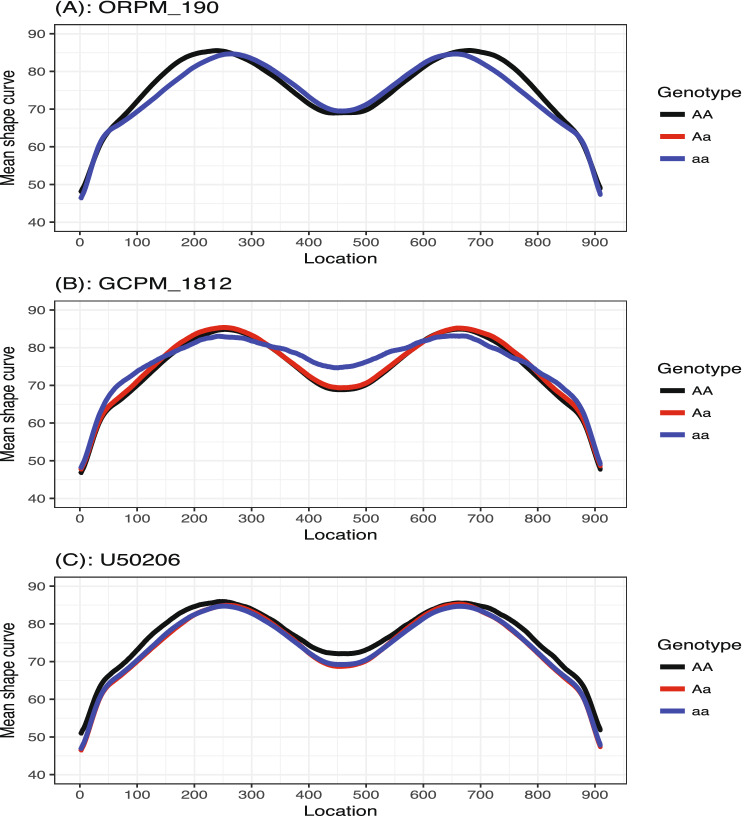
The average of the predicted shape curves output from the FunFor approach across all observations belong to each genotype category for the top three most important markers *ORPM_190*, *GCPM_1812*, and *U50206*. The black curve for AA, the red curve for Aa, and the blue curve for aa.

**Figure 7:**
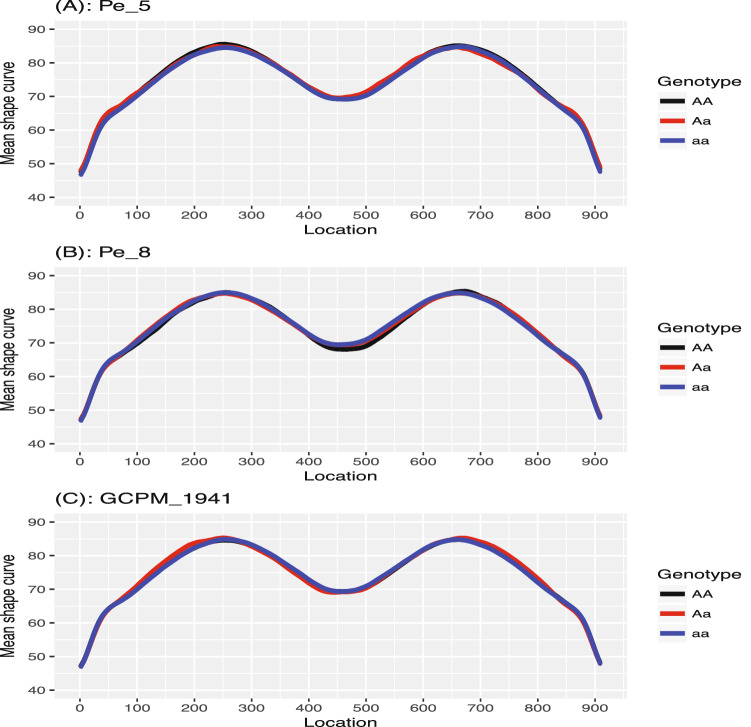
The average of the predicted shape curves output from the FunFor approach across all observations belong to each genotype category for the three least important markers *Pe_5*, *Pe_8*, and *GCPM_1941*. The black curve for AA, the red curve for Aa, and the blue curve for aa.

**Figure 8:**
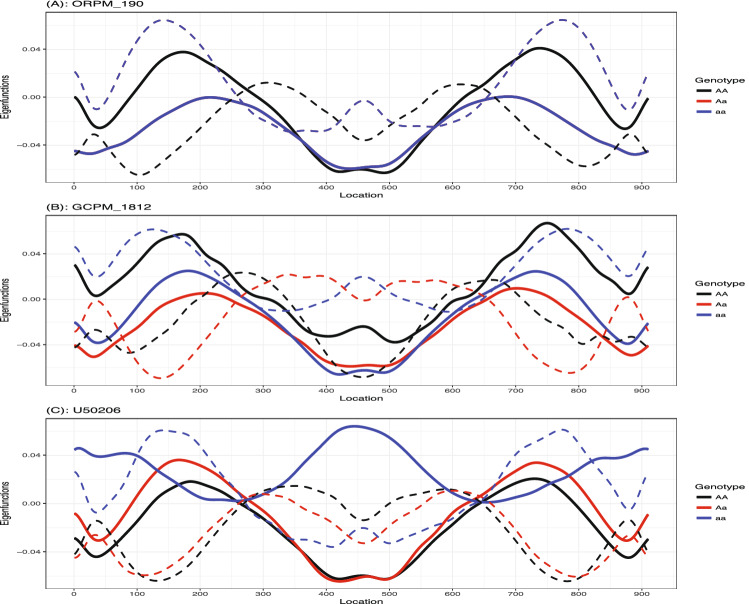
The first two Eigenfunctions output from the FunFor approach across all observations belong to each genotype of the top three most important markers *ORPM_190*, *GCPM_1812*, and *U50206*. The black curve for AA, the red curve for Aa, and the blue curve for aa. Solid line for the first Eigenfunction ${\hat{v}}_{1}(t)$ and the dash line for the second Eigenfunction ${\hat{v}}_{2}(t)$.

Figure 5 demonstrates the PVIMs of all of the genetic markers. The majority of markers have PVIMs close to zero, which reflects the sparsity phenomenon of the genetic dataset. The three markers strikingly standing out with the highest PVIMs ($>200$) are *ORPM*$\_$*190*, *GCPM*$\_$*1812*, and *U50206*, highlighted by red dots. In order to demonstrate the genetic effects of these three important markers, Fig. 6 visualizes the average of the predicted shape curves (i.e., $\bar{{\hat{f}}}_i(t)$) that FunFor outputs across the observations of each genotype category. The three genotype categories exhibit quite different curve trajectories, which indicate the non-negligible association existing between these three genetic markers and the shape curve. Specifically, the marker *ORPM_190* has only two genotypes (*AA* & *aa*) and its red line (corresponding to Aa) does not show up. For marker *GCPM_1812*, the entire trajectories of aa (blue line) and Aa (red line) are very similar, but they are dramatically different from the curve of AA (black line), which represents a recessive genetic effect. For marker *U50206*, the entire trajectories of AA (black line) and Aa (red line) are very similar, but they are different from the curve of aa (blue line), which represents a dominant genetic effect. As a comparison, the results of the three genetic markers (*Pe_5*, *Pe_8*, *GCPM_1941*) with the minimum PVIMs are also visualized in Fig. 7. Opposed to Fig. 6, the three average shape curves corresponding to the three different genotype categories for each of the three markers with minimum PVIMs almost overlap each other. Figure 7 visually reconfirms that the markers with low PVIMs are unlikely to be associated with the responses curve. In addition to the mean shape curve, we also demonstrate the first two eigenfunctions for the three most important markers (see Fig. 8). The first mode accounts for around 41% of the total variation of all shape observations in this data. We noticed that the ${\hat{v}}_1(t)$ for aa (the solid blue line) is completely negative for *ORPM_190*, but completely positive for *U50206*, throughout the entire range. This demonstrates that the same genotype aa of different genetic markers can have opposite contributions to the biggest variability mode among all leaf shapes. The second mode accounts for around 32% of the total variation of all shape observations in this data. We also notice that the signs of the ${\hat{v}}_2(t)$ for aa (dash blue lines) are almost exactly the opposite to those of ${\hat{v}}_2(t)$ for AA (dashed black lines) everywhere among each of the three subplots demonstrated in Fig. 8. This demonstrates that the genotypes aa and AA of the same marker can have opposite contributions to the second mode variation, which corresponds to a measure of uniformity for genotypes of each marker contributing to the shape variation. Actually, Fig. 8 visually confirms that information is lost when independence is assumed or the auto-covariance structure is neglected.

**Figure 9:**
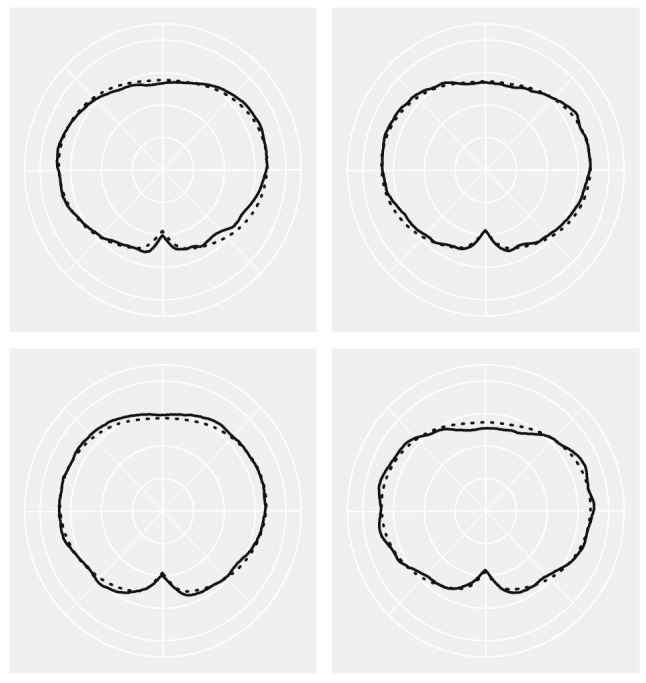
Visualization of the predicted and observed shapes for four observations. The solid lines are the original observed shape (averaging the twenty-five shapes for each observation), and the dot lines are the predicted shape output from the FunFor approach by fivefold cross-validation.

The FunFor can predict the curve response for new observations by following the same tree building and ensemble process if the predictors of new observations are given. To visualize the prediction capability of FunFor approach, we divide our data into training and test sets by fivefold cross-validation. Figure 9 demonstrates that the predicted shapes (dotted lines) and the observed shapes match with great consistency. Note that the observed shape is the one after averaging the twenty-five leaves for each observation, which is the $Y_{ik}$ specified in the model (1).

## Discussion

The FunFor approach extends the well-established traditional random forests approach from a univariate response to a functional curve response, while empowering the traditional functional data analysis approaches with more capabilities to cope with complex structures and nonlinear associations. Most important of all, the proposed FunFor approach has far-reaching applications due to its model-free and distribution-free nature. The real data analysis of this article was performed on a small dataset with a limited number of genetic markers because of the difficulty and cost of collecting the whole genome data for a natural population of trees. These markers did not provide full genome coverage or the concomitant ability to pinpoint specific loci that affect leaf shape traits. However, the proposed FunFor approach is ready to be applied to large datasets. It can be flexibly applied to various fields where the functional data is collected. For example, fMRI, EEG, drug sensitivity curves, climate change, growth curves, and risk prediction in population genetics are possible application directions.

In addition to its aforementioned advantages that are the focus of this article, we now comment on some of the limitations of the FunFor approach as future work directions. This article mainly focused on functional data that is collected at regular and dense time/location points. In future work, we will extend it to be feasible for longitudinal data, i.e., when the data is measured on an irregular grid with various time or location points in the response curve^45–47^. In addition, the FunFor approach adopted the most standard techniques in the forest building process. With the rapid advancement of the forest building process and machine learning skills for the univariate response, the FunFor approach can be improved accordingly in its splitting criteria, variable importance measures, p-values, theoretical inferences, etc.

Specifically, the splitting criterion proposed in Eq. (5) works well in this article; however, alternative splitting criteria can also be tried in the FunFor approach. For example, the splitting function used to measure the within-node homogeneity, defined by $\phi _1(j,s,R)=RSS_f(R)-RSS_f(R_L(j,s))-RSS_f(R_R(j,s))$^22^, or the weighted variance splitting approach that enables a tree to recover from a bad split^48^. The semi-metric proposed by Ferraty and vieu (2006) is an alternative option to replace the $L^2$-metric when we define the proximities between two functional objects^18^. In addition, the permutation variable importance measure that we proposed in Equation (6) may inherit the shortcomings of the traditional random forest and hence yield biased variable selection results or may perturb individual predictors and cause predictions that extrapolate to areas of the predictor space with low density when predictors are dependent^49,50^. Therefore, we will consider incorporating the conditional variable importance measures into the FunFor framework. For example, the conditional predictive impact (CPI) proposed by Watson and Wright was theoretically proven to be a consistent and unbiased estimator^51^; or the model-agnostic variants of the conditional PVIM performed subgroup permutation by constructing subgroups in which the predictor distribution within a group is more homogeneous and between the groups is more heterogeneous^50^. See Degenhardt et al. for a comprehensive review of the several variable selection procedures that have been invented for the traditional random forests for the univariate response^52^. Finally, hypothesis testing has also received great attention in the traditional random forest approach because there is a high need in obtaining p-values to assess the significance levels in biomedical applications. To obtain the p-value for each predictor from the FunFor approach, the nonparametric permutation skill is feasible if the number of predictors is small, as Chen et al. and Hapfelmeier and Ulm did for the traditional random forests approach^53–56^. If the number of predictors is large with sparse structures, we suggest incorporating the FunFor approach with heuristic variable importance test designed for high-dimensional data, which constructs the null distribution based on the non-positive importance scores corresponding to those predictors that are likely non-relevant to the response^57^.

## Supplementary Information

Supplementary Information.
