# Supplementary material for: Functional random forests for curve response
Source: Sci Rep. 2021 Dec 17;11:24159. doi: 10.1038/s41598-021-02265-4 (PMC8683425; doi:10.1038/s41598-021-02265-4)
Supplement: Supplementary file 1 — Supplementary Information. [file 41598_2021_2265_MOESM1_ESM.pdf]

Appendix File for "Functional Random Forests for Curve Response"

August 17, 2021

A Three Extra Simulation Examples

In each of the following simulation examples, we mimic the motivated real dataset, i.e., the gene-shape association. The genetic marker data are coded as 0 for aa or bb, 1 for Aa or Bb, and 2 for AA or BB. Similar to Simulation 1, we set three 360-dimensional curves as the mean responses, $\boldsymbol{\mu_1}$, $\boldsymbol{\mu_2}$, and $\boldsymbol{\mu_3}$, under each of the three genotype categories. These three mean curves are used to describe three true poplar leaves with varying shapes. In these three extra simulations, we set $n = 100$, $p = 100$, and $K = 360$. For the tuning parameters, we set $L = 3$, $ntree = 100$, and $mtry$ is around 40% of the number of predictors, which is close to $p/3$.

A.1 Simulation examples 6-8: a two-way-interaction design

We consider the scenarios in which two true predictors jointly and interactively contribute to the curve response. Marchini et al. (2005) proposed three polygenetic simulation models in human genetics literature and connected the disease status with two genetic markers through the odds ratio of 3×3 combinations of two genetic marker genotypes (see Table S1). They designed three settings to vary the difficulty level of the interaction strengths, respectively. However, Marchini et al. (2005)'s simulation models cannot be directly implemented in our

Table S1: The probability ratio table for Epistasis design in simulations 6-8

Simulation 6	AA	Aa	aa
BB	α	$\alpha(1+\theta)$	$\alpha(1+\theta)^2$
Bb	$\alpha(1+\theta)$	$\alpha(1+\theta)^2$	$\alpha(1+\theta)^3$
bb	$\alpha(1+\theta)^2$	$\alpha(1+\theta)^3$	$\alpha(1+\theta)^4$
**Simulation 7**	**AA**	**Aa**	**aa**
BB	α	α	α
Bb	α	$\alpha(1+\theta)$	$\alpha(1+\theta)^2$
bb	α	$\alpha(1+\theta)^2$	$\alpha(1+\theta)^4$
**Simulation 8**	**AA**	**Aa**	**aa**
BB	α	α	α
Bb	α	$\alpha(1+\theta)$	$\alpha(1+\theta)$
bb	α	$\alpha(1+\theta)$	$\alpha(1+\theta)$

case because their response is binary ($Y = 1$ for disease and $Y = 0$ for non-disease) and hence the response samples can be generated easily once the odds ratio is given. However, our simulation considers three groups; and their response is in categorical type but our simulation considers a continuous high-dimensional curve.

Therefore, we make a few modifications on the designs of Marchini et al. (2005): 1) We extend the traditional logistic model that Marchini et al. (2005) used for generating binary response to a multinomial logistic model to handle three categories. 2) We adjust their odds ratio as the probability ratio of choosing the first shape versus choosing the second or the third shape (p_1/p_2 and p_1/p_3). We use two sets of α's and θ's values, which are chosen close to those suggested by Marchini et al. (2005). Specifically, $\alpha_1 = 0.8$ and $\theta_1 = 0.5$ are used for the p_1/p_2 ratio parameter and $\alpha_2 = 0.9$ and $\theta_2 = 0.4$ are used for p_1/p_3 ratio parameter. Then we generate the two influential genetic markers (call it X_1^* and X_2^*) following a Binomial distribution with a fixed MAF=0.25 and all other noise genetic markers with varying MAF $\sim$ Uniform$(0.1, 0.5)$. Then the curve response samples are simulated through a multinomial logistic regression model with corresponding probabilities, $p(\boldsymbol{Y}_i = \boldsymbol{\mu_1})$, $p(\boldsymbol{Y}_i = \boldsymbol{\mu_2})$, and $p(\boldsymbol{Y}_i = \boldsymbol{\mu_3})$.

Simulation example 6: The baseline is α when the genotype combination is $AABB$, and then it increases multiplicatively $(1 + \theta)$ whenever at least one copy of minor allele a or b appears (see the top penal of Table S1). Since the effects show up in both within and between loci, this model has strong marginal additive effects that are easily detected.

Simulation example 7: The baseline is α when the genotype combination is $AABB$, and then it increases multiplicatively $(1 + \theta)$ when both minor alleles a & b simultaneously appear, additionally it increases another multiplicatively $(1 + \theta)$ whenever one extra minor allele a or b appears (see the middle penal of Table S1). Since the effect of each allele still occurs on some certain conditions, this model is harder to detect compared to the model in Simulation 6.

Simulation example 8: The baseline is α when the genotype combination is $AABB$, and then it increases multiplicatively $(1 + \theta)$ when both minor alleles a & b simultaneously appear, but does not further increase when additional copies of minor allele appear (see the bottom penal of Table S1). Compared to Simulation 7, the power of $(1 + \theta)$ stays the same for four combinations when a & b shows up simultaneously. Since the effect of each observation allele has no role in changing the probability, this model is also harder to detect compared to the model in Simulation 6.

A.2 Simulation results

As shown in Tables S2-3, the FunFor approach performs well in Simulations 6-8. It successfully ranks the two true predictors as the top two ones on average (Table S2); Additionally, the selection is robust among all 100 replications because all different quantiles of M do not show much variation (Table S3), which further indicates that the majority of replications effectively achieve the best results.

Table S2: The average rank of true predictors in Simulations 6-8

Average rank	$Rk^a_{\mathbf{X}^*_1}$	$Rk^a_{\mathbf{X}^*_2}$	Rk^a_{Light}
Simulation 6	1.43	1.62	
Simulation 7	2.77	2.25	
Simulation 8	2.1	3.26	

[a] Rk stands for the average rank of each true predictor across 100 replications. The smaller, the better.

Table S3: Quantiles of the minimum selection size in Simulations 6-8

Minimum selection size	M^b				
Quantiles	5%	25%	50%	75%	95%
Simulation 6	2	2	2	2	2
Simulation 7	2	2	2	3	13
Simulation 8	2	2	2	3	8

[b] M stands for the minimum selection size that is required to include all of the true predictors in each replication. The closer of different quantiles, the robuster.

References

Marchini, J., Donnelly, P., and Cardon, L. R. (2005). Genome-wide strategies for detecting multiple loci that influence complex diseases. *Nature Genetics* **37,** 413–417.
